# Using artificial intelligence to reduce queuing time and improve satisfaction in pediatric outpatient service: A randomized clinical trial

**DOI:** 10.3389/fped.2022.929834

**Published:** 2022-08-10

**Authors:** Xiaoqing Li, Dan Tian, Weihua Li, Yabin Hu, Bin Dong, Hansong Wang, Jiajun Yuan, Biru Li, Hao Mei, Shilu Tong, Liebin Zhao, Shijian Liu

**Affiliations:** ^1^School of Medicine, Shanghai Children’s Medical Center, Child Health Advocacy Institute, Shanghai Jiao Tong University, Shanghai, China; ^2^School of Public Health, Shanghai Jiao Tong University, Shanghai, China; ^3^Division of Hospital Management, Shanghai Children’s Medical Center, Shanghai Jiao Tong University School of Medicine, Shanghai, China; ^4^Pediatric AI Clinical Application and Research Center, Shanghai Children’s Medical Center, Shanghai, China; ^5^Shanghai Engineering Research Center of Intelligence Pediatrics (SERCIP), Shanghai, China; ^6^Department of Pediatric Internal Medicine, Shanghai Children’s Medical Center, Shanghai Jiao Tong University School of Medicine, Shanghai, China; ^7^Department of Data Science, School of Population Health, University of Mississippi Medical Center, Jackson, MS, United States; ^8^School of Public Health and Social Work, Institute of Health and Biomedical Innovation, Queensland University of Technology, Brisbane, QLD, Australia

**Keywords:** artificial intelligence, queueing time, waiting time, satisfaction, randomized controlled trial

## Abstract

**Introduction:**

Complicated outpatient procedures are associated with excessive paperwork and long waiting times. We aimed to shorten queuing times and improve visiting satisfaction.

**Methods:**

We developed an artificial intelligence (AI)-assisted program named *Smart-doctor*. A randomized controlled trial was conducted at Shanghai Children’s Medical Center. Participants were randomly divided into an AI-assisted and conventional group. *Smart-doctor* was used as a medical assistant in the AI-assisted group. At the end of the visit, an e-medical satisfaction questionnaire was asked to be done. The primary outcome was the queuing time, while secondary outcomes included the consulting time, test time, total time, and satisfaction score. Wilcoxon rank sum test, multiple linear regression and ordinal regression were also used.

**Results:**

We enrolled 740 eligible patients (114 withdrew, response rate: 84.59%). The median queuing time was 8.78 (interquartile range [IQR] 3.97,33.88) minutes for the AI-assisted group versus 21.81 (IQR 6.66,73.10) minutes for the conventional group (*p* < 0.01), and the AI-assisted group had a shorter consulting time (0.35 [IQR 0.18, 0.99] vs. 2.68 [IQR 1.82, 3.80] minutes, *p* < 0.01), and total time (40.20 [IQR 26.40, 73.80] vs. 110.40 [IQR 68.40, 164.40] minutes, *p* < 0.01). The overall satisfaction score was increased by 17.53% (*p* < 0.01) in the AI-assisted group. In addition, multiple linear regression and ordinal regression showed that the queuing time and satisfaction were mainly affected by group (*p* < 0.01), and missing the turn (*p* < 0.01).

**Conclusions:**

Using AI to simplify the outpatient service procedure can shorten the queuing time of patients and improve visit satisfaction.

## Introduction

With China’s population exceeding 1.4 billion and the government initiating the *Three-child policy* (a couple can have up to three children), the population boom has spurred an explosion in outpatient visits to pediatric hospitals (1, 2). Long waiting time, crowded waiting areas, and mistrust between doctors and patients have attracted widespread public attention (3, 4). These problems not only seriously influence patients’ satisfaction with doctors and hospitals but also puts constraints on the development of the medical and health industry (5). The long hospital queuing time in China stem from specific outpatient service procedures (6, 7), as most patients do not make an appointment and they can register directly when arriving at the hospital (8). Consequently, they are obliged to line up several times to register, sign in, and see a doctor. Similarly, they will also wait to have an exam, pay the bill, receive the report, and wait again to see their doctor. There appear to be many queuing segments in large hospitals in particular. Also, as far as we know, the longest line is at the door of doctor’s offices (9). Patients clog there in droves and wait for their number to be announced, while worrying they missed the turn and must queue up again if they left for a while.

Domestic and foreign studies describe arrangements taken to reduce queuing time. Some of these arrangements include a cellphone calling system, doctor education, and patient triage (10–12). Others bring telemedicine and online registration with appointment into play (13–15). While reducing queuing time of patients, these methods may also potentially saddle doctors with additional burdens (16). Few studies have actually restructured outpatient service procedures, such as a virtual doctor to interview patients in the field of general surgery department (17, 18). In addition, rare interventions have been conducted exclusively in children’s hospitals, and people do not pay enough attention to common internal medicine diseases.

**FIGURE 1:**
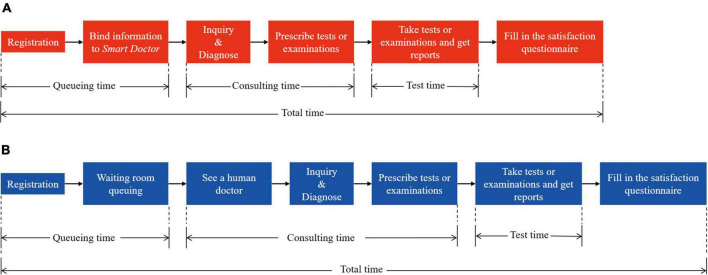
Patient visiting process of conventional group and AI-assisted group. **(A)** AI-based outpatient visiting process. The patient is diagnosed by *Smart-doctor*, which will also prescribe examinations/tests for the patient. **(B)** Conventional outpatient visiting process. The patient is diagnosed and prescribe examinations/tests by a real human doctor.

In our previous retrospective cohort study (19) we introduced *Smart-doctor*, an AI-based medical assistant, into the section for internal medicine department. *Smart-doctor* can model itself on doctors’ reasoning and decision-making processes, treat many patients simultaneously, and prescribe appropriate tests/examinations for them. With its help, patients do not have to queue up to see a human doctor. All patients need to scan a specific two-dimensional code on their cellphones, and then *Smart-doctor* will perform an inquiry. After tests/examinations, patients still need to wait for a clinical doctor to view the reports and then decide what to do next (Figure 1). Given the limited quality of retrospective studies in terms of data loss, logic error, and selection bias therefore, we debugged the hospital information system. Then, we carried out a randomized controlled trial (RCT) to investigate the effect of *Smart-doctor* on queuing time and satisfaction scores of the patients with respiratory and gastrointestinal diseases.

## Methods

### Study design and participants

The study was undertaken at the tertiary hospital of Shanghai Children’s Medical Center (SCMC), affiliated with the School of Medicine at Shanghai Jiao Tong University, which has an inpatient capacity of 1,000 beds. In 2018, our hospital launched *Smart-doctor* in the departments of internal medicine, respiratory medicine, and gastroenterology. In 2019 and 2020, the annual outpatient visits were 1,673,650 and 1,125,699, respectively. Of these, 90.32% visits were treated on the conventional track, and 9.68% visits were treated on the AI-assisted track. *Smart-doctor* is an AI assistant based on a machine learning algorithm that was described in our previous study and in Liang’s study. It was jointly developed by YI TU Technology Co., Ltd. (19, 20).

*Smart-doctor*, an intelligent system based on deep learning-driven natural language processing (NLP) model, recommends which examinations/tests needed to be done before seeing the doctor just like a real doctor’s clinical reasoning process. The study was conducted following the Consolidated Standards of Reporting Trials guidelines (CONSORT, (21). The trial was reviewed by the Institute Review Board of SCMC and registered in ClinicalTrials.gov (Identifier: NCT04186104).

Participants were recruited in the department of internal medicine from August 17, 2020, to April 30, 2021. Uniformly trained investigators enrolled children aged 2 months to 18 years old whose parents consented to their participation. All children were accompanied by their parents during their visit. To be eligible, children were required to have the chief complaint of cough, diarrhea, urination pain, or vomiting. Exclusion criteria were that participants were eliminated if their guardians did not agree to join the study, or their guardians had difficulty in continuing the study. The sample size was estimated according to the results of the preliminary experiment through *PASS* 16.0 (NCSS LLC, Kaysville, Utah, United States). The parameters were based on the results of the preliminary experiment (α = 0.05, statistical power = 0.90, average queueing time of AI-assisted group = 18.84 min, average queueing time of conventional group = 25.38 min, standard deviation of queueing time of AI-assisted group = 19.44 min, and standard deviation of queueing time of conventional group = 26.74 min).

### Intervention and grouping

Enrolled participants were randomized into two arms of the study: participants interviewed by *Smart-doctor* (AI-assisted group, intervention) and participants interviewed by human physicians (conventional group, control). If the patient missed a round, according to the rules of the queue management system (22), they usually had to wait for two more initial patients and one more returning patient before obtaining another round. Patients in both groups had to wait outside the doctor’s office after receiving the reports of the examinations or tests. During this period, if there was a strong willingness of the patient to change groups or withdraw, the outpatient number had to be recorded as a proposal violation (PV). If the patient’s condition became deteriorated, the investigator would communicate with the medical staff in time to let the patient receive timely treatment. During the queuing time, investigators would instruct the guardian of the children to fill in the electronic satisfaction questionnaire.

### Randomization and blinding setting

Eligible participants were randomized into two groups with an allocation ratio of 1:1. SPSS was used to set a fixed value of 20200806 and generate a total of 800 random numbers from 0 to 100. Patients with odd numbers were assigned to the conventional group, and patients with even numbers were assigned to the AI-assisted group.

The blinding method was unsuitable for this study. Although interventions were randomly assigned, specific interventions needed to be implemented by the patients (or their guardians). The vast majority of guardians had the experience of going to a public hospital, and the outpatient procedure in almost all public hospitals in China was generally similar, which was equivalent to our conventional procedure. Therefore, it was not practical to hide the patients or their guardians based on the group to which they were assigned to.

### Satisfaction investigating and data collecting

Satisfaction was investigated using an electronic questionnaire. This questionnaire referred to a longer questionnaire from the study done in Chinese secondary and tertiary hospitals (23). The full satisfaction questionnaire comprised the 8 domains that have been found to be the most important according to the patient’s perception of quality of care: waiting time, autonomy, continuity, efficiency, effectiveness, knowledge, information, and empathy. The reliability and validity of this questionnaire had been verified in Chinese population. As all the patients were minors, the questionnaire was filled out by their guardians. The satisfaction questionnaire consisted of two sections. The first section collected general information, including outpatient ID, way of registration (online/in hospital), whether appointment was made, whether pre-diagnosis was made, whether pre-inquiry was made, as well as the reasons for visiting (chief complaint). The second section investigated parent’s attitudes toward visiting, including registration, pre-inquiry, pre-test, queuing time, service by doctor and other medical staff, experience throughout the visiting, and the need to implement *Smart-doctor* in the hospital. The questionnaire was scored on the Likert scale, including five options for each entry: very satisfied (5 points), relatively satisfied (4 points), average (3 points), dissatisfied (2 points), and very dissatisfied (1 point). When patients did not receive one of these services, we asked the guardians to choose the average (3 points) option.

Data associated with outpatient procedures, including children’s general information (such as age, gender, way of registration and treatment) and timelines (such as registration time, inquiry time, and chargeable time), were captured in the Hospital Information System (HIS). When recruiting patients, investigators recorded the patient’s visit time and visit number to match them with the data in the HIS.

### Evaluating performance of smart-doctor

With the intention of proving the accuracy of *Smart-doctor* in prescribing tests/examinations and enhancing the trust of doctors and guardians in AI-assisted inquisition, we conducted an evaluation study before this trial. We obtained the data of patients in the internal medicine department of SCMC between August 2019 and January 2020 from Electronic Health Records (EHRs). Patients diagnosed with respiratory disease and gastrointestinal diseases were finally selected. The reason for the selection was that these two kinds of diseases had the largest number of patients in the department of general internal medicine. In addition, the condition of such patients was relatively mild, which was more suitable for AI-assisted processing. Guideline for the Diagnosis of Respiratory Diseases in Children and Guideline for the Diagnosis of Gastrointestinal Diseases in Children were considered as the diagnosis standards. We invited three expert doctors to evaluate the items recommended by *Smart-doctor* according to the gold standards. The included cases were evaluated by the first and second specialists respectively. If the two results were consistent, they will be adopted. If they were inconsistent, the results will be determined after discussion with a third expert. Unqualified recommendation of tests/examinations included missed or superfluous tests/examinations. Missed recommendation represented ≥1 item was forgotten to recommend by AI. Superfluous recommendation meant that ≥1 extra and unnecessary item was recommended by AI. We calculated the accuracy of *Smart-doctor* in the prescription for these two types of diseases. And accuracy is a proportion of correct prescriptions (accuracy: correct prescriptions/total prescriptions).

### Outcomes and statistical analysis

The primary outcome was the queuing time, which was the time from registration to the patient’s first entry to the doctor’s office. Secondary outcomes included the consulting time (time taken by doctors to inquire, palpate, and prescribe), testing time (time spent by the patient to receive auxiliary examinations or tests), and total time (time spent by patients from registration to filling out the satisfaction questionnaire). Other outcomes included the comparison of satisfaction scores between two groups, and the factors affecting queuing time and satisfaction. The application of *Smart-doctor* in the pre-test might have adverse consequences, that is, the nimiety, error, or omission of tests/examination. Moreover, in this study, adverse outcomes were indirectly assessed by comparing the percentage of twice prescription of doctor or *Smart-doctor* and the cost between the AI-assisted and the conventional group. Twice prescription means that during the first inquiry, the patient was prescribed with incomplete tests/examinations items. That is to say, some items might be missing. This was because the doctor/AI neglected to prescribe the tests/examinations items, or the doctor/AI prescribed again based on the report of the patient’s first tests/examinations.

Data were screened for normality using the Shapiro-Wilk test. First, we described participant characteristics using Mean ± SD (standard deviation) and proportions, and utilized the t-test or Chi-square test to evaluate the characteristics of patients. Second, we compared the queuing time, consulting time, testing time, and total time by the Wilcoxon rank sum test. Third, we calculated the average scores for each entry in the satisfaction questionnaire and then contrasted the results using the Wilcoxon Rank Sum Test. Fourth, we assessed the association between the variables (group, gender, arriving on time or missing the turn, etc.) and the queuing time using Multiple linear regression (MLR). Fifth, we utilized ordinal regression to analyze the factors (group, queuing time, arriving on time or missing the turn, etc.) that influence the satisfaction score. Sixth, the Chi-square test and the Wilcoxon rank sum test were applied to compare the percentage of twice prescription and the cost between the two groups. Statistics were considered significant at *p* < 0.05 and 1-β > 0.80. Data were analyzed with SPSS 25.0 (IBM, Chicago, Illinois, United States). ITT (Intention-to-Treat), AT (As-Treated), and PP (Per-Protocol) data sets were analyzed.

## Results

### Evaluation of smart-doctor’s performance

The EHRs of 7,725 patients with respiratory diseases and 2,118 patients with gastrointestinal diseases were gathered for the evaluation. According to the assessment of human physicians, if the testing/examination of a patient issued by *Smart-doctor* was in complete agreement with guidelines, the prescription would be defined as correct. Guidelines for the diagnosis of respiratory diseases and gastrointestinal diseases in children were regarded as the gold standards. The accuracy in respiratory diseases was 0.92, and the accuracy in gastrointestinal diseases was 0.85.

### Flow chart of the randomized controlled trial

**FIGURE 2:**
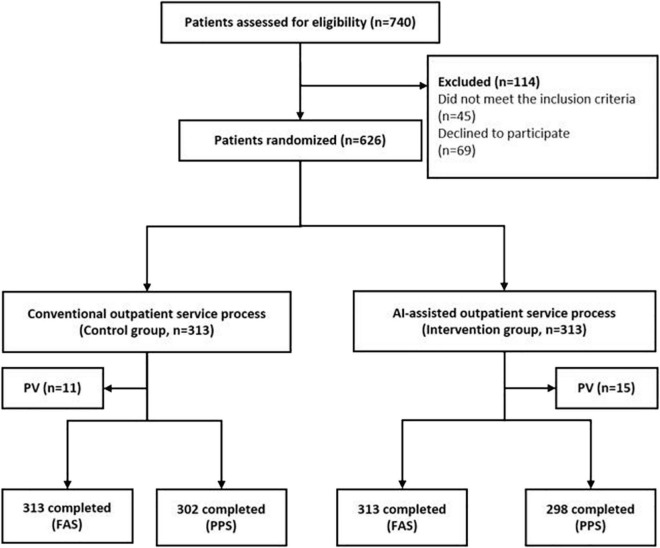
Flow chart of the randomized controlled trial. PV, protocol violation. FAS, full analysis set. PPS, per protocol set.

The sample size was estimated to be 540. Considering reject of participants, we increased the sample size by 10%, and the final sample size was estimated to be 594. During the study period, 740 patients were recruited, and 626 (84.59%) were eligible for randomization (Figure 2). However, 69 parents declined to participate owing to unfamiliar operations of cell phone and temporary situations. Distrust was also a core reason for midway quitting, as *Smart-doctor*’s application time was relatively short and population reach was narrow. Additionally, 45 patients who had complicated concomitant symptoms did not meet the inclusion criteria and were excluded. Of those randomized, 626 (100%) completed the patient satisfaction survey. Eleven patients who had been assigned to the conventional group violated the proposal and were transferred to the AI group, and another 15 patients in the AI-assisted group were transferred to the conventional group according to their requests. Ultimately, 626 patients were included in the Full Analysis Set (FAS) and applied ITT analysis (313 in the AI-assisted group and 313 in the control group. Then, 610 participants were classified into the Per-Protocol Set (PPS) and performed AT analysis (298 in the AI-assisted group and 302 in the control group).

### Basic characteristics of participants

No significant difference was found in age between the AI-assisted and conventional groups (4.05 ± 3.17 vs 4.36 ± 3.42, *p* = 0.241). Table 1 shows the baseline characteristics of the participants. There were 159 (51.02%) boys and 168 (52.60%) boys in the two groups, respectively (*p* = 0.522). We recruited patients strictly according to their chief complaints, and finally included 232 cases with diarrhea, 215 with cough, 146 with urination pain, and 33 patients with vomiting. The Chi-square test showed that patients’ chief complaints did not differ by group (*p* = 0.821). The differences between the control and intervention group were mainly reflected in the registration method, appointment, pre-inquiry, missing their turn, and weekdays. In the AI-assisted group, more people registered through mobile phones, made appointments in advance, used the pre-inquiry service, missed their turn, and visited on weekdays during outpatient service (*p* < 0.01).

**TABLE 1:** Characteristics of participants.

Characteristics	AI-assisted group *N* = 313(%)	Conventional group *N* = 313(%)	Total *N* = 626	*P*
**Gender**				
Male	159 (51.02)	168 (52.60)	327	0.522^a^
Female	154 (48.98)	145 (47.40)	299	
**Self-reported symptom**				
Nausea and vomiting	19 (6.07)	14 (4.47)	33	0.821^a^
Cold and Cough	105 (33.55)	110 (35.14)	215	
Abdominal pain and diarrhea	115 (36.74)	117 (37.38)	232	
Frequent urination urgency	74 (23.64)	72 (23.00)	146	
**Registration way**				
Mobile phone	115 (36.74)	58 (18.53)	173	<0.01^a^
Machine & service window	198 (63.26)	255 (81.47)	453	
**Appointment or not**				
Yes	104 (33.23)	54 (17.25)	158	<0.01^a^
No	207 (66.13)	259 (82.45)	466	
Missing	2 (0.64)	0 (0.00)	2	
**Pre-inquiry or not**				
Yes	194 (61.98)	65 (20.77)	259	<0.01^a^
No	119 (38.02)	248 (79.23)	367	
**Missing the turn or not**				
Yes	30 (9.58)	90 (28.75)	120	<0.01^a^
No	283 (90.42)	223 (71.25)	506	
**Weekdays or weekends**				
Weekdays	267 (85.30)	210 (67.09)	477	<0.01^a^
Weekends	46 (14.70)	103 (32.91)	149	

AI, Artificial intelligence.

^a^Chi-square test.

### Comparison of time and cost between AI-assisted group and conventional group

During the intervention, some guardians rejected the original allocation plans. Therefore, three data sets of ITT, PP, and AT were used. Table 2 displayed the time-related outcomes between the two groups with ITT analysis. Time variables were found significantly to be more reduced in the AI-assisted group than in the control group at time outcomes, including queuing time (8.78 [3.97,33.88] vs. 21.81 [6.66,73.10] min, *p* < 0.01), consulting time (0.35 [0.18,0.99] vs. 2.68 [1.82, 3.80] min, *p* < 0.01), and total time (40.20 [26.40, 73.80] vs. 110.40 [68.40, 164.40] min, *p* < 0.01). In addition, no difference existed in test/examination time between the AI-assisted and conventional group (18.92 [11.10, 30.16] vs. 17.93 [13.19, 26.87] min, *p* = 0.874). Supplementary Tables 1,2 presented the respective results of PP and AT analysis, which were consistent with the results of ITT analysis (Table 2).

**TABLE 2:** ITT analysis of time between AI-assisted group and conventional group.

Time variables	AI-assisted group (*N* = 313) Median (P_25_,P_75_)	Conventional group (*N* = 313) Median (P_25_,P_75_)	*P*
Queueing time, min^a^	8.78 (3.97, 33.88)	46.10 (17.53, 87.79)	<0.01^e^
Consulting time, min^b^	0.35 (0.18, 0.99)	2.68 (1.82, 3.80)	<0.01^ e^
Test time, min^c^	18.92 (11.10, 30.16)	17.93 (13.19, 26.87)	0.874^ e^
Total time, min^d^	40.20 (26.40, 73.80)	110.4 (68.40, 164.40)	<0.01^ e^

ITT, Intention-to-treat. AI, Artificial intelligence. IQR, Inter Quartile range.

^a^Queueing time: The time between the registration and seeing the doctor.

^b^Consulting time: The patient is in the doctor’s consulting room, and the doctor gives the time to inquire, palpate and make out the prescription.

^c^Test time: The time taken for a patient to undergo lab tests/image examinations.

^d^Total time: The time between the patient enters the hospital and leaves the hospital.

^e^Wilcoxon rank sum test.

There was no difference in the percentage of twice prescription between the two groups, with 3.83% in the AI group vs 6.07% in the conventional group (*p* = 0.269). Moreover, patients in the AI-assisted group spent less on test fees (116.72 ± 113.66 vs. 143.16 ± 159.41, *p* < 0.01), examination fees (10.65 ± 39.43 vs. 29.26 ± 82.97, *p* < 0.01), and drug fees (27.15 ± 88.64 vs. 126.30 ± 138.69 *p* < 0.01) than those in the conventional group.

### Satisfaction score between AI-assisted group and conventional group

**FIGURE 3:**
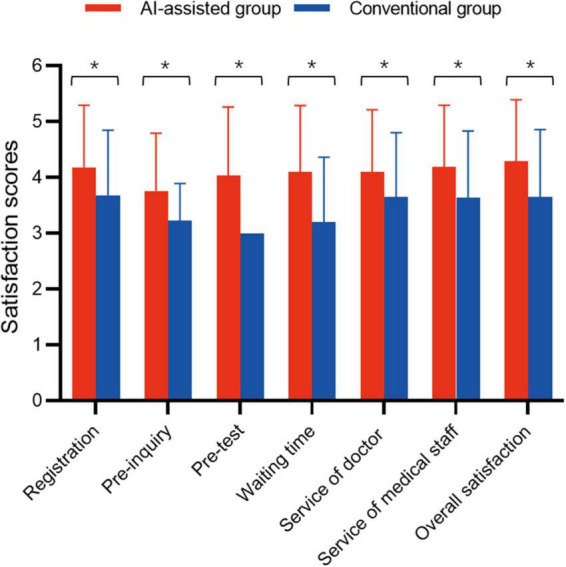
Guardian’s satisfaction scores. *Represents a statistically significant difference. Because the conventional group did not receive a pre-test/examination, the default option was set as “normal” (3 points). Therefore, the scores of the AI-assisted and the conventional group were not compared.

Figure 3 demonstrates the satisfaction scores between the AI-assisted group and conventional group. In the seven items of satisfaction evaluation, the average score of each item in both groups exceeded 3 points. In the satisfaction of registration, pre-inquiry, waiting time, service of doctor or other medical staff, and overall satisfaction, the satisfaction scores in the AI-assisted group were all higher than those in the control group (*p* < 0.01). The greatest difference was the satisfaction score of waiting time; the AI-assisted group improved by 1 point over the conventional group (*Z* = –9.052, *p* < 0.01). As the conventional-group patients did not receive an AI-assisted pre-test ahead of consulting, the default score of this item was set as 3. Thus, we did not assess the pre-test between the two groups.

### Liner regression of queueing time

The results of the multiple linear regression for factors associated with queuing time are presented in Supplementary Table 3. Group, gender, missing the turn or not, weekdays or weekends, symptom, appointment or not, and pre-inquiry or not were independent variables, which were considered likely to influence the queuing time. Model 1 was adjusted for symptom and pre-inquiry or not. We found that a longer queuing time was associated with boys (β = 8.460, *p* = 0.029), missing the turn (β = 45.629, *p* < 0.001), and weekdays (β = 9.184, *p* = 0.048), while the AI-assisted group (β = –28.924, *p* < 0.001) was associated with a shorter queuing time. Moreover, model 2 was adjusted for age, symptom, registration method, pre-inquiry or not, and appointment or not. We found that a longer queuing time were correlated with boys (β = 8.445, *p* = 0.030), missing the turn (β = 45.440, *p* < 0.001), and weekdays (β = 9.302, *p* = 0.046), while the AI-assisted group (β = –29.105, *p* < 0.001) may have lessened the queuing time.

### Ordinal regression of satisfaction score

We established two models to analyze the factors affecting the satisfaction score (Supplementary Table 4). Group, age, gender, pre-inquiry or not, symptom, queuing time, missing the turn or not, and weekdays or weekends were deemed as independent variables, which probably influenced satisfaction. Model 1 was adjusted for age, gender, pre-inquiry or not, and symptom. The results showed that the satisfaction score was significantly associated with queuing time (OR = 0.991 [95% CI: 0.988, 0.994], *p* < 0.01) and weekends (OR = 1.563 [95% CI: 1.106, 2.209], *p* = 0.009). Furthermore, model 2 was adjusted for age, gender, pre-inquiry or not, and symptom. The AI-assisted group (OR = 1.750 [95% CI: 1.256, 2.439], *p* = 0.01) and missing the turn (OR = 0.670 [95% CI: 0.454,0.988], *p* = 0.043) were significantly associated with satisfaction scores.

## Discussion

This study was conducted in Shanghai Children’s Medical Center, affiliated with Shanghai Jiao Tong University, a representative specialized children’s hospital. The innovation of this study lies in exploiting AI to order tests/examinations ahead of doctors’ inquisition.

As for queuing time, there could be various influencing factors. In terms of multiple linear regression, grouping and whether patients missed the turn had the principal impact. The group of patients determined whether they needed to queue up to see the doctor and whether there was an AI assistant in the consultation. On account of the *Smart-doctor*, the queuing time was shortened. The extension of queuing time owing to round missing stemmed from personal reasons. About one-third of patients had problems with missing the turn and spending more time queuing. In addition, the queuing time on weekends was shorter than that on weekdays. This may have been a consequence of fewer patients visiting the hospital on weekends. In China, few staff have to work in hospitals on weekends or holidays, so people are accustomed to having outpatient service on weekdays without appointments. Gender factors may have also impinged on queuing time. From the results of Supplementary Table 3, boys were related to a longer queuing time. However, there is no reasonable explanation for this phenomenon.

We found the satisfaction score of each item in AI-assisted group was significantly higher than that of the conventional group in Figure 2. In our early speculation, the rise in satisfaction may have been related to a shorter queuing time for patients in the AI-assisted group, and the increase in guardians’ satisfaction with doctors and other staff, perhaps because more attention was paid to them in the AI-assisted group. On account of using AI-assisted procedure, they more likely called for staff guidance. Accordingly, in Supplementary Table 4, the model 1 of ordinal regression further confirmed that the less queuing time, the higher satisfaction (β = –0.009, OR = 0.991 95% CI [0.988, 0.994], *p* < 0.01). However, in model 2, when we replaced the variable of queuing time with the group of patients, we found that the correlation between the grouping factor and satisfaction (β = 0.560, OR = 0.573 95% CI 1.750 [1.256, 2.439], *p* < 0.01) was stronger than that between the queuing time. These results might suggest that reducing waiting time is not likely, by itself, to influence satisfaction with hospital service (24). Thus, we suspect that there are several reasons. First, during the implementation of the intervention, patients were not and could not be blinded to the way they were going to be treated, which may have led to bias (25, 26). Second, patients in the AI-assisted group did not know exactly how to use *Smart-doctor*. This was where we needed our investigators to help. However, patients in the conventional group were undoubtedly familiar with procedures, so the investigators may have paid less attention to them. Therefore, improvement in patient satisfaction may have been due to increased attention from medical staff rather than simply reduction in time, just like the Hawthorne Effect (27, 28). Finally, our intervention focused on service completion and did not provide patients with other information, such as test results or causes for delays in care. Interventions that provide more comprehensive information have been associated with improving patient satisfaction (29, 30).

In the previous verification of *Smart-doctor*, the accuracy was about 0.92. Considering that there was also a doctor to review the items to avoid omission and excessive, we found that AI-assisted inquisition could behave well in outpatient service. The verification also provided the basis for large-scale use of *Smart-doctor* in patients. In this study, the adverse consequence was considered as the nimiety, error, and omission of tests/examinations, and we utilized the rate of twice prescription and the cost in outpatient service to evaluate it. Actually, the results showed that both the AI-assisted group and the conventional group may have a situation where the doctor did not prescribe enough tests/examinations at first, but would prescribe them when patients received their reports and went to see the doctor again. However, the difference between the two groups was not statistically significant. The costs of the AI-assisted group were significantly lower than those of the conventional group, indicating that the patients were not given more examinations/tests. To improve the acceptance of guardians, we actually limited the test items that *Smart-doctor* could prescribe in our study. This was reflected in the fact that *Smart-doctor* could only prescribe simple, low-cost, and less invasive items, such as routine bloodwork and abdominal ultrasounds. This was also because the patients we included all had common diseases, and the basic items were sufficient to meet the needs. From these results, *Smart-doctor* was almost as good as a real doctor.

As *Smart-doctor* was applied for a short time, and other domestic hospitals had no similar application, patients were not familiar with the pre-diagnosis test. Therefore, we recruited patients to complete the pre-diagnosis test in the hospital during the study. In fact, patients were not required to do the pre-test inside the hospital. As the system was online, as long as the medical card was bound, the AI-assisted inquisition could inquire about patients without any place and time limitation. Thus, the corresponding prescription would be generated, and patients could directly go to the hospital for tests/examinations. In this way, we believe that AI-assisted inquisition can reduce the burden on hospitals by moving some of the steps that may be done outside the hospital. Also, regarding the severity of COVID-19, the nosocomial transmission of the disease has become a significant link. The crowded queue in the clinic area can increase the risk of infection. However, the use of *Smart-doctor* can reduce the number of people waiting for treatment in the hospital, which can reduce nosocomial transmission of COVID-19 during the pandemic. This sets a good example for other children’s hospitals. Our research was carried out in the department of pediatrics, and the diseases we targeted were the most common diseases. Because we believe that in today’s hospital of highly specialized division, what AI technology needs to undertake is tedious, repetitive work. Patients with common colds and coughs, for example, often need only a routine blood test before their doctor can prescribe follow-up medications. However, for patients with complex and rare diseases, patients can even take a more roundabout course owing to the misdiagnosis of AI. Under the circumstances, we should use AI more carefully.

The current study had several strengths, previously, retrospective cohort studies had been conducted, which provided a basis and experience for this RCT. During implementation, we strictly defined inclusion and exclusion criteria and randomly assigned patients to interventions or control groups, thus reducing selection bias. In addition, the main results were consistent with the retrospective cohort study and our expected results. Prior to the study, we also conducted a clinical registry to guide the RCT. A major limitation of this study was that because the study was conducted at a children’s hospital, the outpatient procedure was almost entirely led by parents. Therefore, the factors affecting queuing time and satisfaction not only depended on the children, but also on the parents’ educational level, income, and occupation. Unfortunately, parents were not cooperative enough when filling out the questionnaire because they were anxious about their children’s illness. Thus, we simplified the form to reduce the time for parents to fill it out. Therefore, the subsequent multi-factor analysis could not explore the influence of parental factors on queuing time and satisfaction. Another limitation of the study stemmed from inclusion criteria. Patients had chief complaints of cough, diarrhea, urination pain, or vomiting, which were mild and relatively common diseases. In fact, from the chief complaint, these patients were with digestive, urinary or respiratory diseases. This was because the contents of prescriptions that Smart-Doctor could prescribe were limited in advance, so we also limited the conditions of patients included. We excluded patients with complex symptoms, which may have led to some selection bias (31). Beyond these, intelligent systems can only solve problems based on evidence. Doctors have difficulty dealing with non-evidence-based systems or with rare cases.

## Conclusion

AI-assisted inquisition is of great value and should be applied to other hospitals, especially in China, where patients with common diseases can register and see a doctor at home with AI-assisted inquisition. Therefore, the resources are more prone to difficult and complicated patients, facilitating reasonable allocation of medical resources. In the near future, we plan to expand Smart-doctor to other departments at SCMC. At the same time, we will introduce AI-assisted inquisition to other children’s hospitals in Shanghai and analyze the effect of Smart-doctor from the perspective of a multi-center study.

## Data availability statement

The raw data supporting the conclusions of this article will be made available by the authors, without undue reservation.

## Ethics statement

The studies involving human participants were reviewed and approved by Ethics Committee of Shanghai Children’s Medical Center. Written informed consent to participate in this study was provided by the participants’ legal guardian.

## Author contributions

XL, SL, and YH drafted the manuscript. DT, WL, BD, HW, JY and BL conducted the statistical analysis. LZ, ST, and HM critically reviewed the manuscript. SL have full responsibility to interpret. All authors approved the final manuscript as submitted and agreed to be accountable for all aspects of the work.
