# Supplementary material for: Using artificial intelligence to reduce queuing time and improve satisfaction in pediatric outpatient service: A randomized clinical trial
Source: Front Pediatr. 2022 Aug 10;10:929834. doi: 10.3389/fped.2022.929834 (PMC9399636; doi:10.3389/fped.2022.929834)
Supplement: Supplementary file 1 [file Table_1.DOCX]

**Table S1** Per-protocol analysis of time between AI-assisted group and conventional group

| **Time variables** | **AI-assisted group (N=298)**  **Median (P_25_,P_75_)** | **Conventional group (N=302)**  **Median (P_25_,P_75_)** | ***p*** |
| --- | --- | --- | --- |
| Queueing time, min ^a^ | 7.76 (3.91, 23.55) | 47.48 (19.81, 88.59) | <0.01^e^ |
| Consulting time, min ^b^ | 0.32 (0.18, 0.84) | 2.68 (1.82, 3.82) | <0.01 ^e^ |
| Test time, min ^c^ | 18.87 (10.92, 28.48) | 17.92 (13.20, 26.80) | 0.806 ^e^ |
| Total time, min ^d^ | 38.11 (25.32, 62.68) | 110.69 (70.67, 166.06) | <0.01 ^e^ |

ITT, Intention-to-treat. AI: Artificial intelligence. IQR, Inter Quartile range. ^a^ Queueing time: The time between the registration and seeing the doctor. ^b^ Consulting time: The patient is in the doctor's consulting room, and the doctor gives the time to inquire, palpate and make out the prescription. ^c^ Test time: The time taken for a patient to undergo lab tests/ image examinations. ^d^ Total time: The time between the patient enters the hospital and leaves the hospital. ^e^ Wilcoxon rank sum test.

**Table S2** As-Treated analysis of time between AI-assisted group and conventional group

| **Time variables** | **AI-assisted group (N=317)**  **Median (P_25_,P_75_)** | **Conventional group (N=309)**  **Median (P_25_,P_75_)** | ***p*** |
| --- | --- | --- | --- |
| Queueing time, min ^a^ | 7.93 (3.98, 23.68) | 52.28 (20.42, 91.17) | <0.01^e^ |
| Consulting time, min ^b^ | 0.33 (0.18, 0.95) | 2.68 (1.82, 3.98) | <0.01 ^e^ |
| Test time, min ^c^ | 18.88 (11.10, 29.18) | 18.33 (13.28, 29.30) | 0.533 ^e^ |
| Total time, min ^d^ | 39.10 (25.93, 63.01) | 113.58 (71.30, 170.27) | <0.01 ^e^ |

ITT, Intention-to-treat. AI: Artificial intelligence. IQR, Inter Quartile range. ^a^ Queueing time: The time between the registration and seeing the doctor. ^b^ Consulting time: The patient is in the doctor's consulting room, and the doctor gives the time to inquire, palpate and make out the prescription. ^c^ Test time: The time taken for a patient to undergo lab tests/ image examinations. ^d^ Total time: The time between the patient enters the hospital and leaves the hospital. ^e^ Wilcoxon rank sum test.

**Table S3** Liner regression of queueing time

| **Models** | **Variables** | **Unstandardized** | | **Standardized *β*** | **t** | ***p*** |
| --- | --- | --- | --- | --- | --- | --- |
|  |  | ***β*** | **Standard error** |  |  |  |
| 1 ^a^ | Conventional group | Reference |  |  |  |  |
|  | AI-assisted groups | -28.924 | 4.358 | -0.261 | -6.638 | <0.001 |
|  | Girl | Reference |  |  |  |  |
|  | Boy | 8.460 | 3.870 | 0.076 | 2.186 | 0.029 |
|  | Arriving on time | Reference |  |  |  |  |
|  | Missing the turn | 45.629 | 5.062 | 0.325 | 9.015 | <0.001 |
|  | Weekends | Reference |  |  |  |  |
|  | Weekdays | 9.184 | 4.635 | 0.071 | 1.981 | 0.048 |
| 2 ^b^ | Conventional group | Reference |  |  |  |  |
|  | AI-assisted groups | -29.105 | 4.418 | -0.263 | -6.587 | <0.001 |
|  | Girl | Reference |  |  |  |  |
|  | Boy | 8.445 | 3.880 | 0.076 | 2.177 | 0.030 |
|  | Arriving on time | Reference |  |  |  |  |
|  | Missing the turn | 45.440 | 5.077 | 0.323 | 8.951 | <0.001 |
|  | Weekends | Reference |  |  |  |  |
|  | Weekday | 9.302 | 4.653 | 0.072 | 1.999 | 0.046 |

^a^ Adjusted for symptom and pre-inquiry or not. ^b^ Adjusted for age, symptom, way of registration, pre-inquiry or not and appointment or not.

**Table S4** Ordinal regression of parent satisfaction scores

| **Models** | **Variables** | ***β*** | **Standard error** | ***p*** | **OR (95% CI)** |
| --- | --- | --- | --- | --- | --- |
| 1 ^a^ | Queueing time | -0.009 | 0.002 | <0.01 | 0.991 (0.988, 0.994) |
|  | Arriving on time | Reference |  |  |  |
|  | Missing the turn | -0.025 | 0.2093 | 0.906 | 0.976 (0.647, 1.471) |
|  | Weekdays | Reference |  |  |  |
|  | Weekends | 0.447 | 0.101 | 0.011 | 1.563 (1.106, 2.209) |
| 2 ^b^ | Conventional group | Reference |  |  |  |
|  | AI-assisted group | 0.560 | 0.1694 | 0.001 | 1.750 (1.256, 2.439) |
|  | Arriving on time | Reference |  |  |  |
|  | Missing the turn | -0.401 | 0.1983 | 0.043 | 0.670 (0.454, 0.988) |
|  | Weekdays | Reference |  |  |  |
|  | Weekends | 0.302 | 0.1776 | 0.089 | 1.353 (.955, 1.917) |

CI, Confidence interval. ^a^ Adjusted for age, gender, pre-inquiry or not, and symptom. ^b^ Adjusted for age, gender, pre-inquiry or not, and symptom.
